# Retinal Nerve Fiber Layer Thickness and Associations With Cognitive Impairment in Parkinson’s Disease

**DOI:** 10.3389/fnagi.2022.832768

**Published:** 2022-02-10

**Authors:** Zihan Chang, Fen Xie, Hualing Li, Feilan Yuan, Lina Zeng, Lin Shi, Shuzhen Zhu, Xiaohe Lu, Xiaobo Wei, Qing Wang

**Affiliations:** ^1^Department of Neurology, Zhujiang Hospital, Southern Medical University, Guangzhou, China; ^2^Department of Ophthalmology, Zhujiang Hospital, Southern Medical University, Guangzhou, China; ^3^Department of Imaging and Interventional Radiology, The Chinese University of Hong Kong, Hong Kong, Hong Kong SAR, China

**Keywords:** Parkinson’s disease, cognitive impairment, retinal nerve fiber layer, indicator, optical coherence tomography

## Abstract

**Objective:**

This study intended to investigate whether retinal nerve fiber layer (RNFL) thickness could become a potential marker in patients with Parkinson’s disease with cognitive impairment (PD-CI).

**Methods:**

Fifty-seven PD patients and 45 age-matched healthy controls (HCs) were recruited in our cross-sectional study and completed optical coherence tomography (OCT) evaluations. PD with normal cognition (PD-NC) and cognitive impairment (PD-CI) patients were divided following the 2015 Movement Disorder Society criteria. RNFL thickness was quantified in subfields of the 3.0-mm circle surrounding the optic disk; while a battery of neuropsychiatric assessments was conducted to estimate the Parkinsonism severity. General linear models and one-way ANOVA were adopted to assess RNFL thickness between subgroups with different cognitive statuses; logistic regression analyses were applied to determine the relation between RNFL and PD-CI cases.

**Results:**

Compared with HCs, more thinning of the RNFL was observed in the inferior and temporal sectors in PD patients, especially in the PD-CI group. Inferior RNFL thickness was reduced in PD-CI compared with PD-NC patients. Logistic regression analysis found that inferior RNFL thickness was independently associated with PD-CI cases (odds ratio = 0.923, *p* = 0.014). Receiver operating characteristic analysis showed that the RNFL-involved combined model provided a high accuracy in screening cognitive deficiency in PD cases (area under the curve = 0.85, *p* < 0.001).

**Conclusion:**

Reduced RNFL thickness especially in the inferior sector is independently associated with PD-CI patients. Our study present new perspectives into verifying possible indicators for neuropathological processes or disease severity in Parkinsonians with cognitive dysfunction.

## Introduction

Parkinson’s disease (PD) is a highly prevalent neurodegenerative disorder worldwide with characteristic motor dysfunctions including bradykinesia, stiffness and resting tremor (Tolosa et al., 2021). Ours and other studies have indicated that some indicators such as neuroinflammatory, neuroimaging mediators and serum neurofilament light chain are usually applied as markers in diagnosing and evaluating the progression and severity of PD and PD Syndromes (Wang et al., 2020, 2021; Yang et al., 2020; Zhu et al., 2020, 2021a; Bestwick et al., 2021; Liu et al., 2021; Quadalti et al., 2021). Identifying potential biomarkers for early diagnosis and assessment is one of the research hotspots in the field of PD (Avenali et al., 2020; Papuć and Rejdak, 2020; Ma et al., 2021). Interestingly, recent increasing evidence indicates that dopamine (DA), as a neurotransmitter, plays a critical role in the retina for visual processing (Archibald et al., 2009; Tsokolas et al., 2020), and previous studies found phosphorylated or misfolded alpha-synuclein accumulation in retinal cells and progressive retinal degeneration in PD patients. These findings imply that structural or functional deficiency of the retina may reflect pathological deterioration in the brain, and visual dysfunctions, especially retinal impairment, in PD might be associated with disease severity and reflected in neuropathogenesis. Whether retinal changes in PD could be used as a potential marker to evaluate severity remains unknown.

Previous studies have demonstrated that multiple disease-specifically structural changes have been verified in the retinas of patients with neurological diseases, for instance, Huntington’s disease, Alzheimer’s disease (AD) and PD. Optical coherence tomography (OCT), a well-developed imaging tool, offers unique high-definition transverse scanning and stereoscopic volume measures of the retina. Several lines of evidence have shown that retinal thickness assessment could provide multiple parameters in distinguishing healthy subjects from those with neurodegenerative diseases (Tsokolas et al., 2020). Previous studies also revealed that the retinal nerve fiber layer (RNFL), macula and fovea thickness in PD cases were markedly reduced compared with that of healthy participants (Chrysou et al., 2019; Tsokolas et al., 2020; Zhou et al., 2021). Other studies also described the connection between the RNFL loss and the disease progression (Garcia-Martin et al., 2014; Jimenez et al., 2014), further indicating the potential application of RNFL thickness as a marker in the clinical evaluation of PD.

However, to our knowledge, very few studies have evaluated OCT-based retinal changes in PD patients with cognitive impairment (CI), which is one of the non-motor symptoms in PD at the mid-late stage (Aarsland et al., 2017; Orgeta et al., 2020; Yuan et al., 2020). Exploring potential markers for CI in PD is critical to better comprehend its pathogenesis and monitor disease progression (Schrag et al., 2017; Reyes-Perez and Bandres-Ciga, 2021). Considering that the potential relation between RNFL degeneration and cognitive deterioration in PD remains undiscovered, it would be valuable and fascinating to investigate whether RNFL thickness could become a potential indicator for CI in PD.

In our study, we (1) conducted a well-ordered data collection from PD patients and healthy controls; (2) characterized their OCT examination and undertook cognitive performance; and (3) evaluated the associations between RNFL parameters and Parkinsonian severity. Last, we hypothesized that peripapillary RNFL thinning in a specific sector could be used as a potential indicator for decreased cognition in PD, and the RNFL-involved model might be effective in distinguishing PD cases with normal cognition from participants with poor cognition.

## Materials and Methods

### Study Design and Participants

From July 2020 to May 2021, 57 PD patients and 45 age- and sex-matched healthy controls were prospectively recruited in our cross-sectional observational study from Zhujiang Hospital, Southern Medical University in Guangzhou, P. R. China. The PD patients diagnosed by experienced neurologists specialized in neurodegenerative disorders, met the 2015 Movement Disorder Society criteria (Postuma et al., 2015), and underwent extensive clinical examination. Healthy controls (HCs) lacking a history of neurological or ophthalmological disease were enrolled from the Medical Examination Center of our hospital. The exclusion criteria were listed as below: (1) presence of incapacity owing to neuropsychiatric comorbidities such as severe cerebral ischemia, psychosis, Alzheimer’s disease (AD), multiple sclerosis or epilepsy; (2) presence of a history of eye trauma, ocular surgery, glaucoma, retinopathy, fundus disease, severe ocular media opacity that hampers the acquisition of high-quality OCT images, and other comorbid ophthalmic pathologies that might influence retinal thickness; (3) medical drugs or severe somatic diseases that may interfere with the neuropsychiatric assessment (e.g., malignancy, severe heart failure, end-stage renal disease, severe anemia); and (4) inability to cooperate with researchers and complete the whole study.

Parkinson’s disease patients were categorized into two groups, comprising the PD with normal cognition (PD-NC) and PD with cognitive impairment (PD-CI), on the basis of MDS Task Force criteria for mild cognitive impairment (PD-MCI) and Parkinson’s disease dementia (PDD) and previously published studies (Goldman et al., 2017). In brief, the patients who met clinically established PD diagnosis, presented cognitive decline reported by either the patient, informant or clinician, and impairment on a scale of global cognitive abilities (*i.e.*, the Montreal Cognitive Assessment [MoCA]) without impaired functional independence in daily life were classified as PD-MCI (Litvan et al., 2012); the patients who presented a dementia syndrome developing based on the established PD diagnosis, more than one cognitive domain impairment, functional deficits in daily life, typical cognitive features and behavioral symptoms were classified as PDD (Emre et al., 2007). PD patients who either fulfilled the MDS PD-MCI or PDD criteria were classified together as PD-CI in the current study; PD cases without meeting the MDS PD-MCI criteria and dementia were designated PD-NC.

### Standard Protocol Approvals and Patient Consents

The Ethics Committee of Zhujiang Hospital, Southern Medical University authorized this study (NO: 2020-KY-004-02) and it was performed following the 1999 National Institutes of Health Human Subjects Policies and Guidance and the principles of the Declaration of Helsinki. Every participant was required to provide a written consent for this study to allow investigators to measure their clinical status.

### Clinical Evaluation

All PD patients underwent comprehensive neurological evaluation. The Unified Parkinson’s Disease Rating Scale (UPDRS) and the modified Hoehn & Yahr scale (H&Y) were applied to determine the Parkinsonism severity and progression (Hoehn and Yahr, 1967; Movement Disorder Society Task Force on Rating Scales for Parkinson’s Disease, 2003). The Montreal Cognitive Assessment (MoCA) and Mini-Mental State Examination (MMSE) were applied to estimate the cognitive function of PD patients (Hoops et al., 2009; Dalrymple-Alford et al., 2010). All the above scales were administered in a blinded manner. Information on sex, age, education, levodopa equivalent daily dosage (LEDD), and disease duration was recorded. Specifically, PD cases were categorized into various subtypes in terms of their parkinsonian symptoms: tremor-dominant (TD) subtype, akinetic rigid/postural instability gait difficulty (AR/PIGD) subtype and mixed subtype according to previously published studies (Marras et al., 2020; Tang et al., 2021; von Coelln et al., 2021).

### Optical Coherence Tomography

**FIGURE 1:**
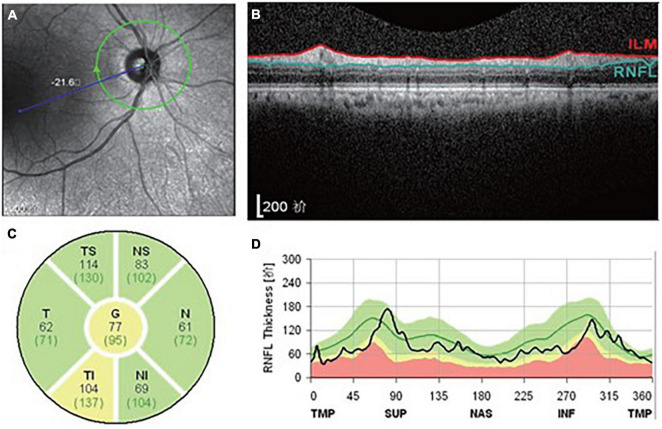
Diagrammatic map revealing the OCT examination of participants in the present study. **(A)** The appearance of the optic nerve. **(B)** Transverse image of RNFL. **(C)** Segmentation of RNFL. **(D)** RNFL thickness map.

We used Spectralis OCT machines in the ophthalmology department of Zhujiang Hospital, Southern Medical University, to obtain the parameters of the peripapillary RNFL. An experienced technician was responsible for performing all the OCT scans and choosing the images of best quality. The diagrammatic map of the OCT examination of participants in the current study is present in Figure 1. The peripapillary region surrounding the optic disk was segmented into four parts, being, superior (S), inferior (I), temporal (T) and nasal (N) sectors. The global (G) RNFL thickness in Figure 1C represents the average thickness of these four sectors. The superior and inferior sectors were further divided into temporal-superior (TS), nasal-superior (NS), temporal-inferior (TI) and nasal-inferior (NI) subregions (Figure 1C). In our study, the RNFL thickness of both eyes in every participant was recorded; then the average of specific RNFL thickness from both eyes of one participant was used in the following analyses.

### Statistical Analysis

Participants with missing necessary data were removed from our study before the final analyses. We conducted a chi-square (χ^2^) test to evaluate differences in gender distribution and one-way analysis of variance (one-way ANOVA) to determine age differences across the three groups (HCs, PD-NC, and PD-CI). Student’s *t* test or Wilcoxon rank-sum/Mann–Whitney *U* test was adopted to compare the differences or distributions of parameters between the PD-NC and PD-CI groups based on whether they met a normal distribution or were ranked data.

For OCT measurements, we performed one-way ANOVA and *post hoc* Bonferroni-corrected analysis to verify differences in RNFL thickness of different sectors among the three groups listed above. A general linear model was used for further identifying adjusted RNFL thickness between PD-NC and PD-CI patients. Logistic regression analyses were conducted to assess the relation between specific RNFL thickness and impaired cognition in PD. E-values were generated from sensitivity analysis to evaluate the confounding by potential uncontrolled or unmeasured confounders (VanderWeele and Ding, 2017). Receiver operating characteristic (ROC) analysis for RNFL and RNFL-related model was performed to further assess the efficacy of RNFL thickness in identifying cognitive deficiency in PD patients. Binary logistic regression analysis and step forward selection of independent variables were applied to produce the combined models, where the dependent variable was group (PD-CI/PD-NC) and the independent variables were potential influencing factors. The Hosmer–Lemeshow test was conducted to assess the model fitness. *P* less than 0.05 was accepted as statistically significant. Statistical Product and Service Solutions 23.0 was applied in all these statistical analyses.

## Results

### Demographic and Clinical Characteristics

The demographic and clinical data are reported in Table 1. Compared with the PD-NC group, the PD-CI patients showed less educated years and more scores of UPDRS-III, MMSE and MoCA (Table 1).

**TABLE 1:** Demographic and clinical characteristics of the study subjects.

Variables ^†^	Controls (*n* = 45)		PD		*p* ^‡^
			
		All (*n* = 57)	PD-NC (*n* = 23)	PD-CI (*n* = 34)	
**Demographic**					
Age, years	61.42 ± 9.75	63.67 ± 9.27	60.52 ± 8.68	65.79 ± 9.18	0.059
Male sex, No. (%)	21 (46.7%)	34 (59.6%)	12 (52.2%)	22 (64.7%)	0.276
Education, years	/	9 (6)	9 (3)	6 (4)	0.009**
**Clinical**					
H&Y staging	/	2.50 (0.5)	2.00 (0.5)	2.50 (1.0)	0.325
Disease duration, months	/	36 (54)	36 (36)	48 (75)	0.189
LEDD, mg	/	511.50 (242.75)	436.50 (461.50)	515.25 (177.00)	0.083
**PD-subtype, No. (%)**					0.857
AR/PIGD subtype		34 (59.6%)	14 (60.9%)	20 (58.8%)	/
TD subtype		7 (12.3%)	2 (8.7%)	5 (14.7%)	/
Mixed subtype		16 (28.1%)	7 (30.4%)	9 (26.5%)	/
**UPDRS-total**	/	46.0 (26.0)	37.0 (26.0)	49.5 (29.0)	0.016*
UPDRS-I	/	4.0 (3.0)	3.0 (3.0)	4.0 (3.0)	0.137
UPDRS-II	/	15.0 (9.0)	13.0 (8.0)	16.0 (9.0)	0.022*
UPDRS-III	/	25.0 (16.0)	22.0 (17.0)	26.5 (19.0)	0.037*
UPDRS-IV	/	3.0 (4.0)	3.0 (4.0)	3.50 (5.0)	0.094
**MMSE**	/	25.0 (4.0)	28.0 (2.0)	24.5 (3.0)	< 0.001***
**MoCA**	/	22.5 (8.0)	24.5 (2.0)	19.5 (7.0)	< 0.001***

***^‡^**The continuous variables are presented as mean ± standard deviation or median with interquartile range according to normality test; the categorical variables are presented with percentages. **^‡^**From Chi-square (χ^2^) test for gender and one-way ANOVA for age differences across three groups (healthy controls, PD-NC and PD-CI); Wilcoxon rank-sum/Mann-Whitney U test for education, H&Y staging, disease duration, LEDD, UPDRS, MMSE and MoCA differences between PD-NC and PD-CI group; Chi-square (χ^2^) test for PD-subtype difference between PD-NC and PD-CI group. PD, Parkinson’s disease; PD-NC, PD with normal cognition; PD-CI, PD with cognitive impairment; LEDD, levodopa equivalent daily dosage; AR/PIGD, akinetic-rigid/postural instability gait difficulty; TD, tremor-dominant; UPDRS: Unified Parkinson’s Disease Rating Scale; H&Y, modified Hoehn and Yahr staging scale; MMSE, mini-mental state examination; MoCA, Montreal Cognitive Assessment.*

### Comparison of OCT Measurements Among PD-NC and PD-CI Patients and HCs

**FIGURE 2:**
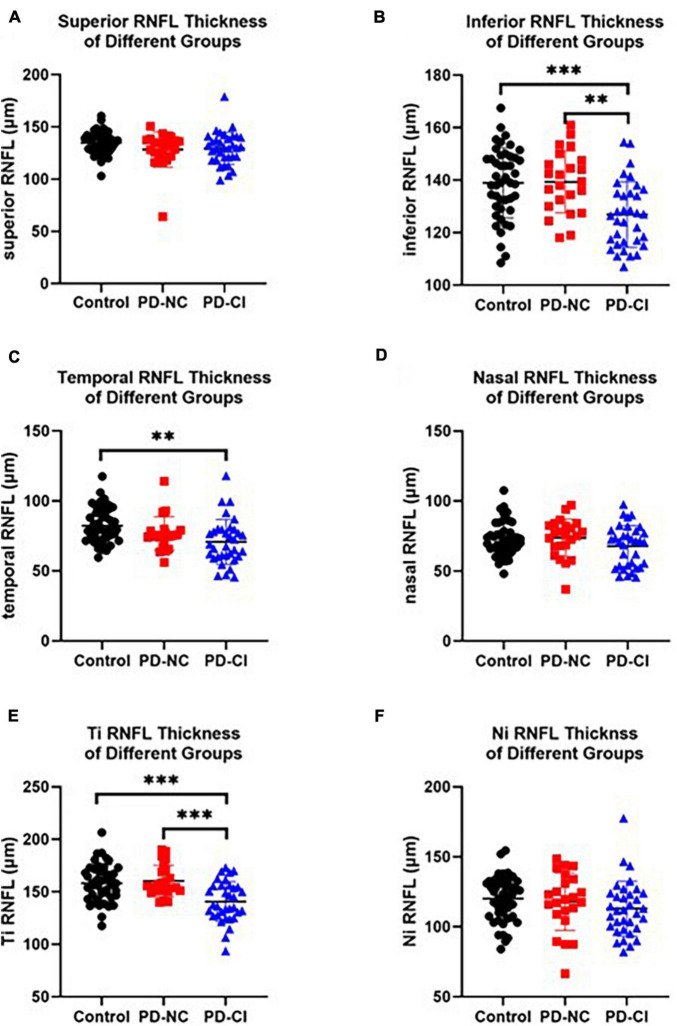
RNFL thickness in PD patients with different cognitive conditions and healthy subjects. The scatter plots accompanied by the mean ± SD present the peripapillary RNFL thickness in healthy controls, PD-NC and PD-CI patients in inferior **(A)**, inferior **(B)**, temporal **(C)**, nasal **(D)**, Ti (temporal-inferior, **E**) and Ni (nasal-inferior, **F**) sectors. **p < 0.01, ***p < 0.001.

One-way ANOVA showed that the thickness of the global RNFL, inferior RNFL and temporal RNFL was markedly reduced in PD-CI cases compared with HCs (Figures 2B,C and Table 2). Only the inferior RNFL showed significant thinning in the PD-CI group compared with the PD-NC group (126.87 ± 12.53 *vs.* 139.33 ± 11.75, *p* = 0.001, Figure 2B and Table 2), indicating a potential specific association between inferior RNFL thickness and Parkinsonian cognitive impairment. Similar findings were identified in the temporal-inferior subregion but not in the nasal-inferior subregion (Figures 2E,F and Table 2).

**TABLE 2:** Comparisons of retinal nerve fiber layer among different groups based on one-way ANOVA.

RNFL (μ m)	*Controls*	*PD*−*NC*	*PD*−*CI*	*P*	*p*	*p*

	Group A	Group B	Group C	A *vs*. B	A *vs*. C	B *vs*. C
Global	107.02 ± 6.55	104.48 ± 9.07	98.50 ± 9.95	0.719	< 0.001***	0.029*
Superior	134.82 ± 10.83	128.37 ± 16.81	129.57 ± 15.42	0.221	0.301	1.000
Inferior	138.98 ± 13.31	139.33 ± 11.75	126.87 ± 12.53	1.000	< 0.001***	0.001**
temporal	82.31 ± 12.65	76.83 ± 12.01	70.78 ± 16.09	0.369	0.001**	0.321
Nasal	73.29 ± 12.14	73.63 ± 13.53	67.79 ± 14.62	1.000	0.217	0.323
**sub-region:**						
temporal-superior	149.04 ± 17.13	142.07 ± 16.15	140.62 ± 20.64	0.412	0.132	1.000
nasal-superior	121.33 ± 16.33	114.52 ± 20.09	117.85 ± 19.49	0.449	1.000	1.000
temporal-inferior	158.22 ± 17.68	160.46 ± 14.94	140.79 ± 18.97	1.000	< 0.001***	< 0.001***
nasal-inferior	120.14 ± 16.22	117.98 ± 20.69	113.01 ± 19.58	1.000	0.276	0.963

* p < 0.05, ** p < 0.01, *** p < 0.001. RNFL: retinal nerve fiber layer; PD: Parkinson’s disease; PD-NC: PD with normal cognition; PD-CI: PD with cognitive impairment.

To further investigate the RNFL thickness in PD patients with different cognitive statuses, we performed general linear model analysis. The thickness of the inferior RNFL and its subregion temporal-inferior RNFL was substantially thinner in PD-CI patients than that of PD-NC group (*e.g*., inferior RNFL**:** 127.75 ± 12.22 *vs.* 138.03 ± 12.41, *p* = 0.005, Table 3) after adjusting for other confounders.

**TABLE 3:** General linear model analysis in the RNFL levels in PD patients with different cognitive status.

RNFL	Global RNFL (μ m)		*p* ^†^	Inferior RNFL (μ m)		*p* ^†^
	PD-NC	PD-CI		PD-NC	PD-CI	
**(A)**						
Unadjusted	104.48 ± 9.07	98.50 ± 9.95*	0.025	139.33 ± 11.75	126.87 ± 12.53***	<0.001
Adjusted	103.16 ± 9.63	99.39 ± 9.48	0.166	138.03 ± 12.41	127.75 ± 12.22**	0.005

**RNFL**	**Temporal-inferior RNFL (μ m)**		***p*** **^**†**^**	**Nasal-inferior RNFL (μ m)**		***p*** **^**†**^**
	**PD-NC**	**PD-CI**		**PD-NC**	**PD-CI**	

**(B)**						
Unadjusted	160.46 ± 14.94	140.79 ± 18.97***	<0.001	117.98 ± 20.69	113.01 ± 19.58	0.363
Adjusted	159.64 ± 18.20	141.34 ± 17.92**	0.001	116.20 ± 21.29	114.22 ± 20.96	0.740

**^**†**^**p values were produced from analysis of covariance, after adjusting for pairwise comparisons. The covariates comprised age, sex, H&Y staging, LEDD, disease duration and PD-subtype. *p < 0.05, **p < 0.01, ***p < 0.001, vs. PD-NC Group.

*Abbreviations: RNFL: retinal nerve fiber layer; PD: Parkinson’s disease; PD-NC: PD with normal cognition; PD-CI: PD with cognitive impairment.*

### Logistic Regression and Correlation Analyses Between RNFL Thickness and Cognitive Impairment

Logistic regression analyses were implemented to further estimate the relation between RNFL thinning and cognitive deficiency in participants with PD. By means of adjusting for possible confounders including sex, age, disease duration, H&Y staging, LEDD, PD subtype, education level, and UPDRS score, inferior RNFL was independently associated with decreased cognition in PD (odds ratio [OR] = 0.923 for every one-micrometer increase, 95% confidence interval 0.865–0.984, *p* = 0.014; Table 4A). Similar findings were confirmed in the logistic regression analysis of temporal-inferior RNFL thickness (Table 4B). In contrast, no such associations were identified between superior, temporal and nasal RNFL thickness and cognitive impairment (Supplementary Table 1). Correlation analysis confirmed a positive correlation between inferior RNFL thickness and MMSE/MoCA scores (Supplementary Table 2).

**TABLE 4:** Logistic regression analysis for the relation between RNFL and the impaired cognition in PD patients.

Global RNFL	Cognitive Impairment (CI)		*p* ^†^	Inferior RNFL	Cognitive Impairment (CI)		*p* ^†^
	OR	95% confidence intervals			OR	95% confidence intervals	
**(A)**							
Unadjusted	0.935*	0.897 – 0.994	0.032	Unadjusted	0.922**	0.877 – 0.970	0.002
Adjusted	0.962	0.893 – 1.037	0.314	Adjusted	0.923*^‡^	0.865 – 0.984	0.014

**Temporal-inferior RNFL**	**Cognitive Impairment (CI)**		***p*** **^**†**^**	**Nasal-inferior RNFL**	**Cognitive Impairment (CI)**		***p*** **^**†**^**
	**OR**	**95% confidence intervals**			**OR**	**95% confidence intervals**	

**(B)**							
Unadjusted	0.932^**^	0.894 – 0.973	0.001	Unadjusted	0.987	0.961 – 1.015	0.358
Adjusted	0.923^**^^§^	0.870 – 0.978	0.007	Adjusted	0.991	0.959 – 1.023	0.558

**^**†**^**the covariates included age, sex, disease duration, H&Y staging, LEDD, PD-subtype, education level, and UPDRS score. **^**‡**^**the E-value generated from sensitivity analysis for OR and upper limit of confidence interval is 1.25 and 1.14, respectively. **§**the E-value for OR and upper limit of confidence interval is 1.25 and 1.17, respectively. *p < 0.05, **p < 0.01. RNFL, retinal nerve fiber layer; PD, Parkinson’s disease; CI, cognitive impairment; OR, odds ratio.

### Discriminative Efficacy of the RNFL-Involved Model in the Identification of Parkinsonian Cognitive Impairment

**FIGURE 3:**
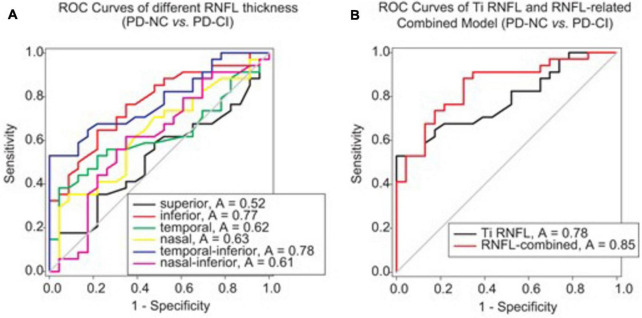
ROC analysis to assess the validity of RNFL thickness or RNFL-involved models for differentiating PD patients with different cognitive conditions. **(A)** The ROC curves of RNFL thickness from different peripapillary sectors are shown. The AUCs of RNFL thickness in the inferior and temporal-inferior sectors were 0.77 (*p* = 0.001) and 0.78 (*p* < 0.001), respectively, for discriminating between PD-NC and PD-CI cases. **(B)** The RNFL-involved combined model for differentiating PD-CI from PD-NC patients comprised three variables, namely Ti (temporal-inferior) RNFL thickness, education level and UPDRS-III. The AUC was 0.85 for this model (*p* < 0.001). Note that the RNFL-involved combined model was generated from the logistic regression analysis in Supplementary Table 3.

ROC analysis was applied to investigate whether RNFL thickness could offer reliable differentiation between PD cases with good cognition and the others with impaired cognition. We found that among the several sectors, only inferior and temporally inferior RNFL thickness exhibited a moderate accuracy in distinguishing PD-CI from PD-NC patients (*e.g.*, AUC of temporal-inferior RNFL thickness: 0.78, *p* < 0.001, Figure 3A; cutoff thickness: 138.8 μm, sensitivity: 52.94%, specificity: 100%).

Then, after stepwise forward selection of demographic, clinical and OCT variables, temporal-inferior RNFL thickness, education level and UPDRS-III were screened out in logistic regression analysis to compose the discriminative model (Supplementary Table 3). This combined model revealed higher accuracy for discrimination than RNFL thickness alone (AUC = 0.85, *p* < 0.001, Figure 3B; cutoff value: 0.56 in the algorithm of the model, sensitivity: 88.24%, specificity: 69.57%).

## Discussion

The present study showed an independent association between peripapillary RNFL thinning and CI in PD. Our findings presented several original observations. First, the RNFL thickness of specific inferior region was declined in PD-CI patients compared with PD-NC subjects by adjusting for confounders, including sex, age, H&Y staging, disease duration, LEDD and PD subtypes. Second, we identified that inferior RNFL thickness was closely associated with CI in PD with logistic regression analysis, independent of various other factors. Third, our study indicated that the RNFL-involved model might be useful to discriminate PD patients with normal cognition from those with impaired cognition. This study suggests that the inferior RNFL thickness may become a possible marker for parkinsonian cognitive impairment.

Recently increasing evidences show that neuroimaging or neuroinflammatory markers are closely related with the pathological neurodegeneration in PD (Lian et al., 2020; Yang et al., 2020; Anandhan et al., 2021; Li et al., 2021; Liu et al., 2021; Que et al., 2021; Wang et al., 2021; Zhu et al., 2021b). These humoral inflammatory or imaging biomarkers may provide valuable information for early identification, differential diagnosis, assessment of severity and even prognosis prediction for PD patients (Avenali et al., 2020; Beheshti et al., 2020). As a window to the brain, the retina may consistently suffer inflammation and degeneration, and provide potential neuroimaging, or, neuroinflammatory markers (Ramirez et al., 2017; Fernández et al., 2021). The uncovering of the disease-specific biomarker α-synuclein accompanied by the retinal progressive degeneration further emphasizes the potential role of the retina in the pathological process or pathogenesis of PD (Adam et al., 2013; Bodis-Wollner, 2013; Bodis-Wollner et al., 2014; Ortuno-Lizaran et al., 2018). One OCT study found thinner RNFL thickness of the inferior quadrant of PD cases than that of healthy subjects, with the inferotemporal area being the thinnest region (Inzelberg et al., 2004). Ahn and his colleague observed RNFL thinning in the inferior and temporal 2.22-mm sectors in drug-naive patients with PD (Ahn et al., 2018). Some meta-analyses further summarized a remarkable loss of the peripapillary RNFL in PD patients, with one article describing an exception for the nasal sector (Chrysou et al., 2019; Zhou et al., 2021). Consistent with previous studies, we found evident peripapillary RNFL thinning particularly in the inferior and temporal sectors in PD cases (Table 2 and Figures 2B,C).

Strikingly, RNFL thinning in the current study was more significant in PD-CI cases than in PD-NC cases (Table 2 and Figure 2), implying that patients with PD-CI may suffer more severe retinal degeneration. General linear model analysis further verified this hypothesis and showed a remarkable decline in the RNFL thickness of the inferior sector and its temporal-inferior subregion in PD-CI subjects compared with the PD-NC subjects (Table 3). Few studies, as we know, have explored OCT parameters especially in individuals suffering from Parkinsonian disease with cognitive impairment. Yıldız et al. described significant parallel association between MMSE-recall score and RNFL-right in PD in a cross-sectional study (Yıldız et al., 2019). Other compelling evidence about OCT investigations on cognitive dysfunction emerged mostly from studies in mild cognitive impairment or AD, demonstrating a reduction of the RNFL thickness surrounding the optic papilla, particularly in the superior and inferior quadrants (Doustar et al., 2017; Kashani et al., 2021). This current study observed a different changing pattern showing a thickness loss in specific inferior RNFL in PD-CI cases (Tables 2, 3 and Figure 2B), implying that the inferior RNFL, to some extent, is more likely to be injured than the other three peripapillary sectors in PD cases with CI. Consequently, the pathological mechanisms underlying the specificity of inferior RNFL thinning in PD patients with CI have become an interesting question.

Evidences from previous studies have indicated that the aggregation of phosphorylated α-synuclein and its subsequent DA loss within the retina may be the primary cause of visual dysfunctions including reduced visual acuity, hallucinations and abnormal spatial contrast sensitivity in individuals with PD (Jackson and Owsley, 2003; Hamedani and Willis, 2020; Mohana Devi et al., 2020). These retinal alterations suggest simultaneous changes in the brain (Kashani et al., 2021). Another study proposed that neurodegenerative pathology in the central nervous system (CNS) may cause retrograde degeneration toward the axon and related soma of retinal ganglion cells (Peng et al., 2020). These studies strongly implied that the specific thinning of the peripapillary RNFL may be triggered by local disease-specific protein inclusions. Similar studies showed that Aβ plaques with concurrent neuronal degeneration were substantial in the superior and inferior retinal quadrants in cases with AD (Koronyo et al., 2017). Since there is a partial overlap of the pathological abnormalities among varieties of neurodegenerative disorders such as AD and PD (Aarsland et al., 2017; Kotagal et al., 2018; Ghadery et al., 2020), the vulnerability of inferior or temporal peripapillary RNFL in PD-CI (shown in Tables 2,3) may suggest more misfolded protein aggregation in these retinal segments, which should be addressed in future studies (Doustar et al., 2017).

Logistic regression analyses were implemented to further investigate the relation between RNFL thickness and cognitive impairment. It showed that inferior and temporal-inferior thickness was associated with decreased cognition in PD, independently of various risk factors for parkinsonian cognitive deterioration including age, sex, H&Y staging, levodopa usage, disease duration, akinetic-rigid/postural instability gait difficulty (AR/PIGD) phenotype, education level and UPDRS score (Table 4; Vasconcellos and Pereira, 2015; Aarsland et al., 2017; Baiano et al., 2020). Meanwhile, inferior RNFL thickness was positively correlated with MoCA and MMSE scores (Supplementary Table 2). Previous studies have implied that the retinal thinning correlated with disease duration or parkinsonian severity as measured by UPDRS or H&Y staging (Garcia-Martin et al., 2014; Jimenez et al., 2014). However, few studies declared the relationship between retinal thinning and parkinsonian cognitive deficiency. Thus, we provided an interesting finding to indicate inferior RNFL thickness as an independent marker in PD-CI patients.

Finally, the discriminative efficacy of retinal thinning for parkinsonian cognitive impairment was investigated. The RNFL thickness of the inferior sector, especially the temporal-inferior subregion, exhibited a moderate accuracy in differentiating PD-CI from PD-NC patients (Figure 3A); the RNFL-involved model revealed a higher accuracy in distinguishing PD-CI from PD-NC patients in comparison to temporal-inferior RNFL thickness alone (Figure 3B) when three variables (i.e., temporal-inferior RNFL thickness, education level and UPDRS-III) were screened out in this model. This implies that even though various demographic and clinical variables have been documented as hazard factors for cognitive deficiency in PD, inferior RNFL thickness could still become a possible indicator for cognitive impairment in PD (Vasconcellos and Pereira, 2015; Guo et al., 2019; Baiano et al., 2020).

The first limitation in our investigation arises from the relatively small sample size, though the statistical power is sufficient. Besides, due to this relatively small sample, we did not specifically divide PD-CI patients into PD-MCI and PDD groups. Thus, the disease-specific changing pattern of RNFL thinning in parkinsonian cognitive deterioration deserves further investigation. Given the trend of inferior RNFL thickness with increasing cognitive impairment in PD at a cross sectional level, longitudinal follow-up is needed to determine inferior RNFL thickness changes over time and to offer insight into predictors of cognitive deterioration in PD. A previous study found the inner retinal layers suffered more obvious thinning in PD patients with long disease duration (Garcia-Martin et al., 2014). Another study reported a regression equation that could predict the UPDRS score with using RNFL thickness as an independent variable (Jimenez et al., 2014). From the perspective of cognition, whether RNFL be used to predict cognitive impairment when a PD patient still has normal cognition at early stage remains an unsolved question. Thus, the advantage and significance of our study is that we first provide additional evidence to show the relation between RNFL thinning and cognitive impairment in PD cases, and further present new perspectives into verifying possible predictors for parkinsonian cognitive deterioration in future longitudinal studies.

## Conclusion

In brief, we confirmed that inferior RNFL thickness was specifically decreased in PD patients with cognitive impairment compared with those with normal cognition; RNFL thinning of the peripapillary inferior sector is a possible indicator of decreased cognition in PD. Our data further indicate the integral roles of retinal degeneration in the pathophysiological mechanisms underlying cognitive deficiency in PD. Large PD cohorts and longitudinal studies are needed to verify our results. Further studies are encouraged to reveal more precise features of peripapillary RNFL thinning in various PD subgroups (e.g., PD-MCI versus PDD; PD-MCI single-domain versus PD-MCI multiple-domain) and PD-plus.

## Data Availability Statement

The raw data supporting the conclusions of this article will be made available by the authors, without undue reservation.

## Ethics Statement

The studies involving human participants were reviewed and approved by The Ethics Committee of Zhujiang Hospital, Southern Medical University. The patients/participants provided their written informed consent to participate in this study.

## Author Contributions

ZC, XW, SZ, and QW conceived and designed the study. ZC, FX, HL, LZ, and FY performed the study. XL and LS revised the article for intellectual content. XW and ZC performed data statistics and analysis. ZC, XW, and QW wrote the article. All authors read and approved the final manuscript.

## Conflict of Interest

The authors declare that the research was conducted in the absence of any commercial or financial relationships that could be construed as a potential conflict of interest.

## Publisher’s Note

All claims expressed in this article are solely those of the authors and do not necessarily represent those of their affiliated organizations, or those of the publisher, the editors and the reviewers. Any product that may be evaluated in this article, or claim that may be made by its manufacturer, is not guaranteed or endorsed by the publisher.
