# Supplementary material for: Retinal Nerve Fiber Layer Thickness and Associations With Cognitive Impairment in Parkinson’s Disease
Source: Front Aging Neurosci. 2022 Feb 10;14:832768. doi: 10.3389/fnagi.2022.832768 (PMC8867012; doi:10.3389/fnagi.2022.832768)
Supplement: Supplementary file 1 [file Table_1.DOC]

**Supplementary Material:**

**Supplementary Table 1.** Logistic regression analysis for the relationship between RNFL and the impaired cognition in PD patients

**Supplementary Table 2.** Correlation analysis of RNFL thickness with demographic/clinical features in PD patients.

**Supplementary Table 3.** Logistic regression analysis to screen demographic, clinical and OCT variables for indicating cognitive impairment in PD patients

**Supplemental Table 1. Logistic regression analysis for the relationship between RNFL and the impaired cognition in PD patients**

(a)

| Superior RNFL | Cognitive Impairment (CI) | | *p* **^†^** |
| --- | --- | --- | --- |
|  | OR | 95% confidence intervals |  |
| Unadjusted | 1.005 | 0.972 – 1.039 | 0.777 |
| Adjusted | 1.031 | 0.977 – 1.089 | 0.266 |

**(**b)

| Temporal RNFL | Cognitive Impairment (CI) | | *p* **^†^** |  | Nasal RNFL | Cognitive Impairment (CI) | | *p* **^†^** |
| --- | --- | --- | --- | --- | --- | --- | --- | --- |
|  | OR | 95% confidence intervals |  |  |  | OR | 95% confidence intervals |  |
| Unadjusted | 0.971 | 0.935 – 1.009 | 0.137 |  | Unadjusted | 0.970 | 0.933 – 1.009 | 0.135 |
| Adjusted | 0.984 | 0.935 – 1.035 | 0.536 |  | Adjusted | 0.979 | 0.929 – 1.032 | 0.434 |

**†**: Potential confounding variables of the models are age, gender, disease duration, H&Y staging, LEDD, PD-subtype, education level, and UPDRS score.

Abbreviations: RNFL: retinal nerve fiber layer; PD: Parkinson’s disease; OR: odds ratio.

**Supplemental Table 2. Correlation analysis of RNFL thickness with demographic/clinical features in PD patients**

|  | age | | disease duration | | H&Y | | MMSE | | MoCA | | UPDRS | |
| --- | --- | --- | --- | --- | --- | --- | --- | --- | --- | --- | --- | --- |
|  | *r*_p_ | *p* | *r*_s_ | *p* | *r*_s_ | *p* | *r*_s_ | *p* | *r*_s_ | *p* | *r*_s_ | *p* |
| superior RNFL | -0.249 | 0.062 | -0.112 | 0.407 | -0.369** | 0.005 | 0.031 | 0.818 | 0.058 | 0.667 | -0.298* | 0.024 |
| inferior RNFL- | -0.265* | 0.046 | -0.147 | 0.276 | -0.417** | 0.001 | 0.361** | 0.006 | 0.433** | 0.001 | -0.327* | 0.013 |
| temporal RNFL | -0.042 | 0.757 | -0.150 | 0.264 | -0.193 | 0.150 | 0.118 | 0.380 | 0.164 | 0.223 | -0.128 | 0.343 |
| nasal RNFL | -0.118 | 0.380 | 0.087 | 0.522 | -0.226 | 0.091 | 0.242 | 0.069 | 0.121 | 0.371 | -0.153 | 0.256 |

***** *r*_p_: Pearson’s correlation coefficients; *r*_s_: Spearman’s rank correlation coefficient.

*: *p* < 0.05, **: *p* < 0.01.

Abbreviations: RNFL: retinal nerve fiber layer; PD: Parkinson’s disease; UPDRS: Unified Parkinson’s Disease Rating Scale; H&Y: modified Hoehn and Yahr staging scale; MMSE: mini-mental state examination; MoCA: Montreal Cognitive Assessment.

**Supplementary Table 3.** Logistic regression analysis to screen demographic, clinical and OCT variables for indicating cognitive impairment in PD patients**^†^**

| Variables in Model ^‡^ | Coefficient | Standard error | Wald χ2 | *p* value^§^ | Odds Ratio | 95%CI for Odds Ratio |
| --- | --- | --- | --- | --- | --- | --- |
| Ti RNFL thickness | - 0.065 | 0.023 | 8.076 | 0.004 | 0.937 | 0.896 - 0.980 |
| UPDRS-III | 0.047 | 0.027 | 2.888 | 0.089 | 1.048 | 0.993 – 1.106 |
| Education level | - 0.160 | 0.085 | 3.577 | 0.059 | 0.852 | 0.721 – 1.006 |
| constant | 10.478 | 3.570 | 8.615 | 0.003 | 35521.261 | / |

Abbreviations: OCT: optical coherence tomography; PD: Parkinson’s disease; Ti: temporal-inferior; RNFL: retinal nerve fiber layer; CI: confidence interval; UPDRS-III: the Unified Parkinson’s Disease Rating Scale, part III.

†: These combined models were generated using binary logistic regression analysis with groups (PD-CI/PD-NC) as the dependent variable and the potential influencing factors listed in Table 4 as independent variables; stepwise forward selection of clinical and biological variables was applied in logistic regression.

‡: *p* = 0.324 for Hosmer-Lemeshow test.

§: Probability for stepwise: Entry: 0.05, Removal: 0.10.
